# Feasibility and accuracy of DireCt Lung Ultrasound Evaluation technique to monitor extravascular lung water in porcine lungs

**DOI:** 10.1093/ejcts/ezae428

**Published:** 2024-12-10

**Authors:** Sana N Buttar, Hasse Møller-Sørensen, Michael Perch, Rene H Petersen, Christian H Møller

**Affiliations:** Department of Cardiothoracic Surgery, Copenhagen University Hospital, Rigshospitalet, Denmark; Department of Clinical Medicine, University of Copenhagen, Copenhagen, Denmark; Department of Cardiothoracic Anaesthesiology, Copenhagen University Hospital, Rigshospitalet, Denmark; Department of Clinical Medicine, University of Copenhagen, Copenhagen, Denmark; Department of Cardiology, Copenhagen University Hospital, Rigshospitalet, Denmark; Department of Cardiothoracic Surgery, Copenhagen University Hospital, Rigshospitalet, Denmark; Department of Clinical Medicine, University of Copenhagen, Copenhagen, Denmark; Department of Cardiothoracic Surgery, Copenhagen University Hospital, Rigshospitalet, Denmark; Department of Clinical Medicine, University of Copenhagen, Copenhagen, Denmark

**Keywords:** Lung transplantation, Extravascular lung water, Cellular *ex vivo* lung perfusion

## Abstract

**OBJECTIVES:**

Extravascular lung water precedes deterioration of pulmonary function. Current tools to assess extravascular lung water in a setting of donor lung procurement and *ex vivo* lung perfusion (EVLP) are either subjective or not feasible. Therefore, a direCt Lung Ultrasound Evaluation (CLUE) has been introduced. This study reassesses the feasibility and accuracy of CLUE by measuring its correlation with lung weight, wet-to-dry ratio (W/D ratio), dynamic compliance and pulmonary vascular resistance (PVR) in a porcine model.

**METHODS:**

CLUE images, lung weight, dynamic compliance and PVR were recorded and lung samples for W/D ratio were taken before and after EVLP. CLUE score was calculated based on B-lines on images taken at each point of the lung using an established equation.

**RESULTS:**

Eighteen porcine lungs were included. Total median of CLUE score, lung weight, W/D ratio and PVR increased significantly, while median of dynamic compliance decreased significantly after EVLP. Total median CLUE score increased significantly in all four surfaces after EVLP with equally high CLUE scores in posterior and diaphragm lines. CLUE score demonstrated a significant strong positive correlation with lung weight (*r* = 0.825) and W/D ratio (*r* = 0.837), while CLUE’s correlation with dynamic compliance and PVR was significantly moderate to strong (*r* = −0.669, *r* = 0.695, respectively).

**CONCLUSIONS:**

CLUE technique is feasible to assess extravascular lung water in donor lungs after procurement and during EVLP. CLUE score correlated significantly with lung weight, W/D ratio, dynamic compliance and PVR. Transplant suitability of a donor lung may not solely depend on CLUE evaluation.

## Graphical abstract

**Figure:**
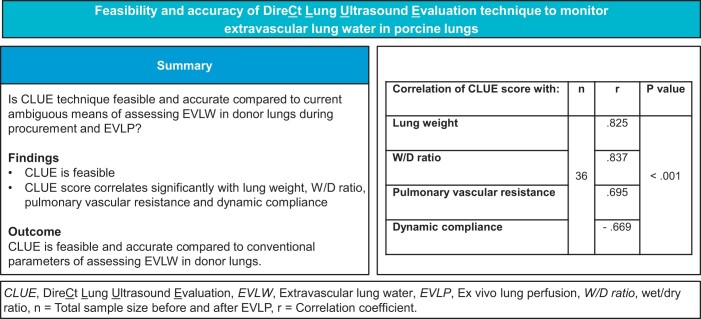

## INTRODUCTION

*Ex vivo* lung perfusion (EVLP) technique assesses and potentially improves marginal donor lungs for lung transplantation (LTx) [1, 2]. One of the main potentials of EVLP is to reduce donor lung edema represented by extravascular lung water (EVLW) [3, 4]. The amount of EVLW in donor lungs is affected by (i) the management of brain- and circulatory-dead donors, (ii) the lung procurement, (iii) the lung preservation and (iv) EVLP [5–9]. A significant presence or increase in donor lung EVLW is known to precede deterioration of pulmonary function [10, 11]. In fact, it has been demonstrated that donor lungs with higher EVLW are more likely to be unsuitable for transplantation and, if transplanted, they would have adverse recipient outcomes [12]. For that reason, the measurement of EVLW in donor lungs is of crucial importance.

Currently, several clinically diagnostic methods are available to assess EVLW including chest X-ray, computed tomography (CT) scan, pulse index continuous cardiac output (PiCCO) technique and a transthoracic lung ultrasound (T-LUS) assessment [13–16]. While chest X-ray has demonstrated a poor correlation with EVLW, CT-scan, PiCCO and T-LUS assessment have shown a high sensitivity for quantification of EVLW with potential to guide clinical management of thoracic and LTx patients [17–20]. Nevertheless, these methods are well established and primarily limited to the settings of either intensive care units or emergency rooms [20, 21]. Thus, diagnostic tools to assess EVLW in donor lungs during the lung procurement phase and in an EVLP setting are relatively unexplored. Few studies have utilized CT scan and PiCCO during EVLP to improve the donor lung evaluation; however, their routine implementation in an EVLP setting is limited due to their inaccessibility and invasive nature for the latter method [14, 22]. Therefore, the current standard EVLW assessment technique of donor lungs remains to be based on either (i) subjectivity (i.e., palpation), (ii) donor lung weight, (iii) assessment of partial pressure of oxygen (PO_2_)/fraction of inspired oxygen (P/F) ratio in addition to conventional lung parameters such as dynamic compliance and pulmonary vascular resistance (PVR) during the EVLP, or (iv) not feasible in the clinical setting [i.e. wet-to-dry (W/D) ratio measurement] [9, 23, 24]. To counter these ambiguous EVLW estimations in addition to reduce EVLW evaluation disparity among clinicians, one centre has introduced a direCt Lung Ultrasound Evaluation (CLUE) technique for EVLW assessment of donor lungs during procurement and EVLP [9, 24, 25]. Based on B-line detection by direct ultrasound on the lung parenchyma, CLUE has been proposed as a feasible and more accurate diagnostic tool for donor lung assessment and management in a setting of donor lung procurement and EVLP [9, 24, 25].

To the best of our knowledge, no other centre has re-evaluated the CLUE technique. This study aims to reproduce the CLUE technique in order to re-assess its feasibility and accuracy by measuring its correlation with lung weight, W/D ratio, dynamic compliance and PVR in a porcine model.

## MATERIALS AND METHODS

### CLUE technique and scoring

**Figure 1:**
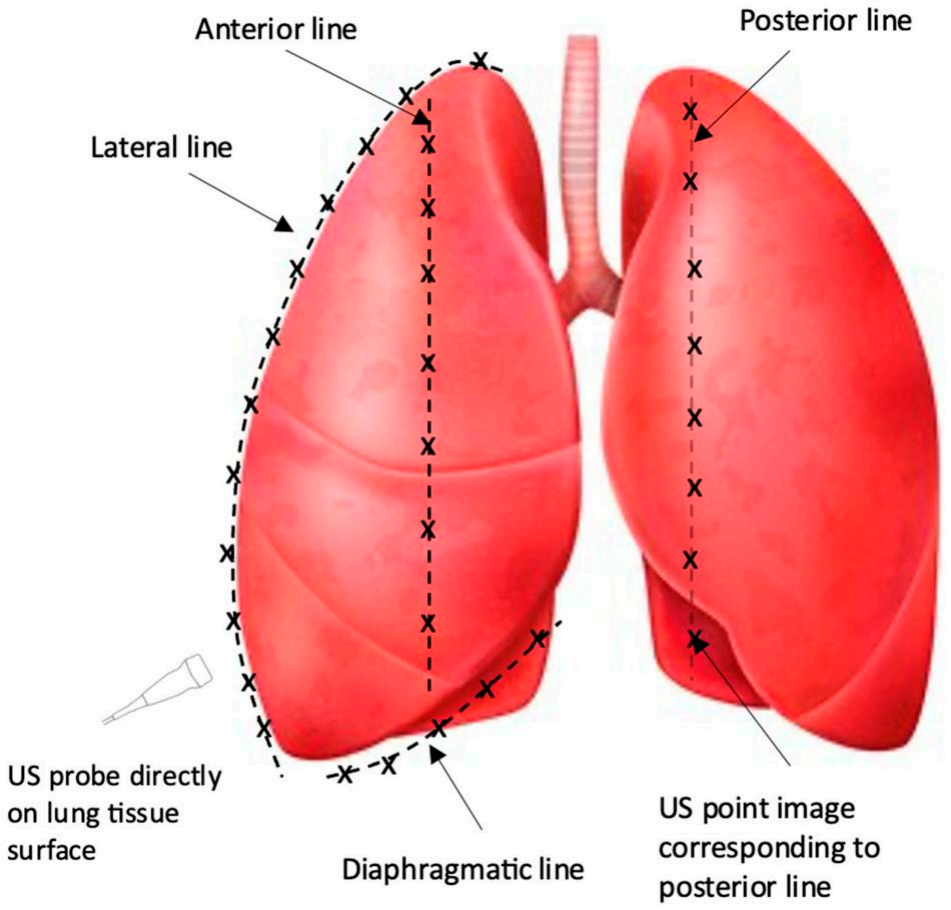
Illustration of the location (represented by dotted lines) and points (represented by the X) of CLUE images. CLUE: DireCt Lung Ultrasound Evaluation; US: ultrasound.

**Figure 2:**
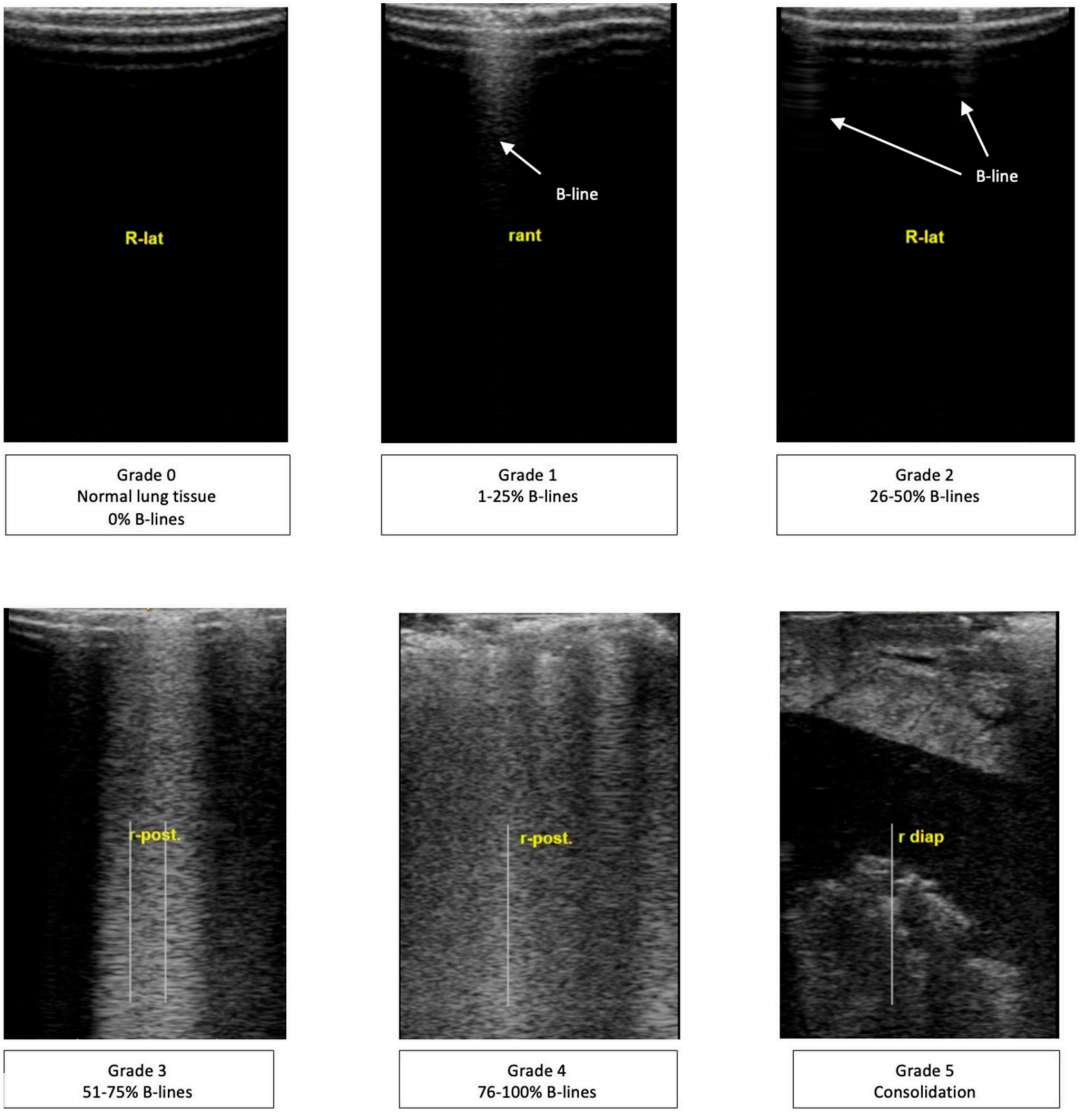
CLUE images for each grade. ant.: anterior; CLUE: DireCt Lung Ultrasound Evaluation; diap: diaphragm surface; lat.: lateral; post.: posterior; R: right lung.

Similar CLUE technique and scoring were used as described by Ayyat *et al*. [9, 24, 25]. In brief, inflated porcine lungs were retrieved and ultrasound images were taken directly from the lung tissue surface at each point of 4 lines passing through the anterior, lateral, posterior and diaphragmatic surfaces (Fig. 1). Images were obtained from each lung with Venue Go R2 (GE Medical System Ultrasound, USA) ultrasound system with a 3.4–12.6 MHz probe. Each ultrasound image was graded from 0 to 5 according to EVLW content based on the percentages of B-lines covering the image (Fig. 2). Based on these grades, a total CLUE score was calculated using following equation: Total CLUE score = [number (no). of grade 1 images × 1 + no. of grade 2 images × 2 + no. of grade 3 images × 3 + no. of grade 4 images × 4 + no. of grade 5 images × 5)/(total no. of images taken].

CLUE was performed before and after the EVLP for each lung side. The investigator (S.N.B) performing CLUE in all lungs was unaware of lung weight, W/D ratio and lung parameters at the time of the CLUE evaluation. To assess the reproducibility of the CLUE score, images were recorded and independently reviewed by 2 evaluators (S.N.B and H.M.S). The inter-class correlation coefficient of each CLUE image rating was found to be 0.86 (Supplementary Material, Appendix A).

## ANIMAL PREPARATION

Healthy domestic female pigs were anaesthetized and mechanically ventilated (Supplementary Material, Appendix B). After sternotomy and main pulmonary artery cannulation, washed red blood cells were collected using a cell-saver. The aorta was clamped upon arrhythmia followed by an immediate—without warm ischaemia period—antegrade flush of 2 l of cold Perfadex (XVIVO Perfusion AB, Gothenburg, Sweden). The lungs were then inflated, trachea was clamped and lungs were harvested with subsequent 2 hours ‘cold ischaemia’ in 4–8°C saline. Lungs were kept inflated during the procurement, cold ischaemia storage and CLUE performance.

### Cellular *ex vivo* lung perfusion procedure

The execution of EVLP protocol has been described in detail previously [26]. In brief, the modified Lund protocol with three different flows [40% (*n* = 6), 80% (*n* = 6) or 100% (*n* = 6)] of estimated cardiac output and intended 8-hour EVLP was used for this experiment. Vivoline LS 1 (Vivoline Medical AB, Sweden) system was primed with STEEN solution (XVIVO Perfusion AB, Sweden) and washed red blood cells (Supplementary Material, Appendix C). Lungs were connected to the circuit with the commencement of antegrade flow of either 40%, 80% or 100% of cardiac output. Ventilation was started at 32°C reaching complete ventilation at 37°C.

### Assessment of lung parameters during *ex vivo* lung perfusion

Dynamic compliance and PVR were recorded every hour during the EVLP. Values ‘Before EVLP’ were taken at hour 0 of EVLP, which was defined as reached the intended full flow rate (40%, 80% or 100% of cardiac output) at 37°C. Values used for ‘After EVLP’ were defined as those recorded at the end of the EVLP.

### Lung weight and wet-to-dry ratio assessment

Lung weight was measured before and after EVLP. For W/D ratio, a 1 × 1 × 1 cm biopsy was taken from one of the corresponding lines used for CLUE images before and after EVLP. The biopsy samples were then frozen at −80°C until the ratio of the weight of the portion before and after drying (60°C in an oven for 24 hours) was calculated.

### Statistical analysis

Continuous data were presented as median (interquartile range), due to the distribution of the variables and compared using Wilcoxon’s test. Spearman’s correlation coefficient (*r*) was used to measure the monotonicity of the relationships between parameters and CLUE score. A *P*-value <0.05 was considered statistically significant. All statistical analyses were performed with RStudio version 2022.07.2.

## RESULTS

**Figure 3:**
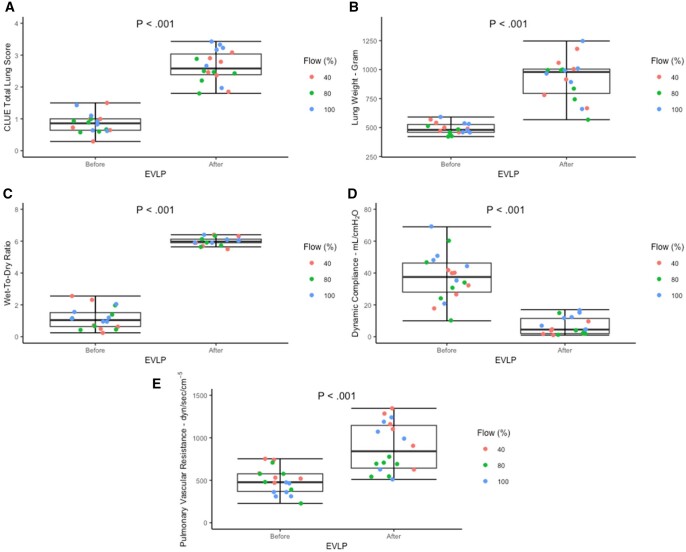
Comparison of (**A**) CLUE total lung score, (**B**) lung weight, (**C**) wet-to-dry ratio, (**D**) dynamic compliance and (**E**) pulmonary vascular resistance before and after the EVLP of all three flow groups. The middle horizontal line represents the median in the bar graphs, and the upper and lower whiskers represent the maximum and minimum values. CLUE: direCt Lung Ultrasound Evaluation; EVLP: *ex vivo* lung perfusion.

Eighteen porcine lungs were assessed on EVLP. Total median of CLUE score, lung weight, W/D ratio and PVR increased significantly, while dynamic compliance decreased significantly after compared to before EVLP (Table 1, Fig. 3A–E). Each variable demonstrated the same significant median trend before and after EVLP for each flow; however, it was insignificant for PVR in 80% of EVLP flow (Supplementary Material, Appendix D). The total median CLUE score increased significantly in all four surfaces after EVLP with posterior and diaphragm surfaces gaining equally high CLUE scores (Table 2). Each line demonstrated the same significant median CLUE score increase in each flow; however, it was insignificant in the anterior line of 80% EVLP flow and the diaphragm line of 40% EVLP flow (Supplementary Material, Appendix E).

**Table 1:** Comparison of parameters ‘Before’ and ‘After’ EVLP

EVLP	Before		After		
	Median	IQR	Median	IQR	*P*-value
CLUE score	0.86	0.64–1	2.58	2.38–3.03	<0.001
Lung weight (g)	480	459–527.5	976	794.8–1004.5	
Wet-to-dry ratio	1.04	0.63–1.51	5.970	5.90–6.12	
Pulmonary resistance (dyn/sec/cm^−5^)	477	369–576	841.5	643–1146	
Dynamic compliance (ml/cmH_2_O)	37.5	28–46.2	4.5	2–11.5	

CLUE: direCt Lung Ultrasound Evaluation; EVLP: *ex vivo* lung perfusion; IQR: interquartile range.

**Table 2:** Comparison of CLUE score between surfaces ‘Before’ and ‘After’ EVLP

EVLP	Before		After		
	Median	IQR	Median	IQR	*P*-value
Anterior surface	1.25	1.1–1.9	2.6	2.1–2.9	<0.001
Lateral surface	1.2	1.1–1.5	2.7	2.3–3.1	
Posterior surface	1.7	1.2–2.1	3.6	2.9–3.9	
Diaphragm surface	1.8	1.1–2.1	3.6	3–4.2	

CLUE: direCt Lung Ultrasound Evaluation; EVLP: *ex vivo* lung perfusion; IQR: interquartile range.

**Figure 4:**
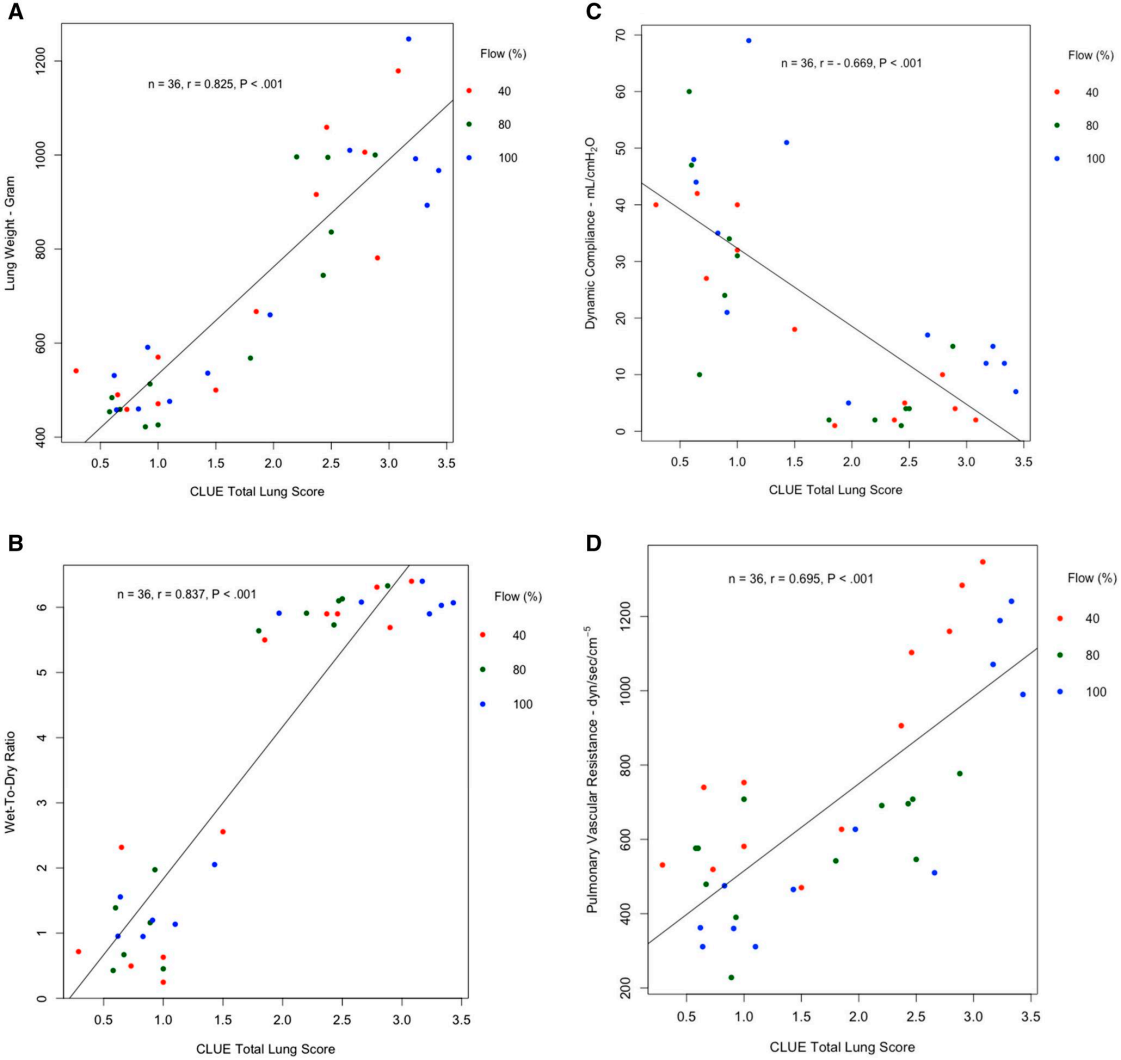
Correlation of (**A**) lung weight, (**B**) wet-to-dry ratio, (**C**) dynamic compliance and (**D**) pulmonary vascular resistance with total CLUE score before and after EVLP of all three flow groups. CLUE: direCt Lung Ultrasound Evaluation; EVLP: *ex vivo* lung perfusion; *n*: total sample size before and after EVLP; *r*: correlation coefficient.

Significantly strong positive correlation was demonstrated between total CLUE score and lung weight and W/D ratio, while CLUE score demonstrated a significantly moderate to strong correlation with PVR (*n* = 36, *r *= 0.825, *P* < 0.001; *n* = 36, *r* = 0.837, *P* < 0.001; *n* = 36, *r* = 0.695, *P* < 0.001, respectively) (Fig. 4A, B, D). Similarly, a significantly moderate to strong but negative correlation was demonstrated between CLUE score and dynamic compliance (*n* = 36, *r* = −0.669, *P* < 0.001) (Fig. 4C). Correlation of CLUE score with each variable in each flow was significantly moderate to strong except PVR of 80% flow (*n* = 12, *r* = 0.529, *P* = 0.07) (Supplementary Material, Appendix F).

## DISCUSSION

T-LUS has the ability to identify B lines, which are increasingly substantiated to have a correlation with EVLW [16, 18–20, 27–29]. As a result, T-LUS has been identified as a relatively accurate adjunct tool to assess EVLW with its additional advantage of being non-invasive and easily accessible in comparison to current clinically methods of EVLW evaluation [30]. Even though T-LUS has primarily been used to monitor respiratory patients, a study has demonstrated its significant role in management of lung donor patients leading to early real-time identification of pulmonary pathology with subsequent prompt intervention resulting in rescue of donor lungs for transplantation [31]. Taking this into consideration, CLUE technique, which is developed from basic principles of T-LUS, is meant to offer the ability of extending donor lung EVLW monitoring non-invasively, easily and accurately during procurement and EVLP [9, 24, 25].

Currently, one of the parameters used for EVLW assessment in donor lungs is lung weight—a method considered to be valid for general quantitative of pulmonary oedema [24]. Although it has been demonstrated to be affected by visceral congestion/blood loss, no relationship has been found between lung weight and body weight/mass index [32]. Similarly, correlation between lung weight and EVLW has been demonstrated to be independent of gender and presented illness [15]. In this study, a positively strong and significant correlation was demonstrated between CLUE score and lung weight suggesting that CLUE score can be equally reliable as lung weight to assess EVLW content in donor lungs. Indeed, this finding is supported by previous CLUE studies thus strengthening the case for CLUE [9, 24]. Although, more studies are required to validate CLUE as a definitive independent evaluator of EVLW, its structured and consistent properties will not only make the decision of donor lung suitability for transplantation uniform across the centres but also omit the subjective bias when assessing a donor lung with current tools including lung weight.

The current gold standard method to evaluate EVLW is W/D ratio [18, 23]. However, this method is invasive requiring lung tissue samples thus not feasible in a clinically setting [18, 23]. Therefore, the clinically feasible gold standard method is PiCCO, which has markedly been correlated with W/D ratio, though with a disadvantage of being invasive and harder to access [33]. Similar latter concern has been raised for the clinical use of CT scan in addition to its radiation related risks, despite its remarkable property of identifying EVLW [19, 34]. Although mounting evidence significantly correlates the T-LUS ability to assess EVLW, it remains inferior to PiCCO and CT scan; however, it is superior in terms of being non-invasive and easily accessible attracting its clinically implementation [14, 18, 20, 29, 34]. Previous studies have used animal models to verify a significant correlation between EVLW measured by W/D ratio and T-LUS [19, 28]. Similarly, patient studies have been used to validate the relationship between EVLW quantified by PiCCO and T-LUS [18, 20, 29]. Thus, the increasing evidence of T-LUS as a satisfactory clinical tool to assess EVLW in a clinical setting [18–20, 28, 29]. In the case of EVLW assessment in donor lungs, porcine models have demonstrated a significant correlation between EVLW measured by W/D ratio and CLUE making CLUE method well-founded in a setting of donor lung procurement and EVLP [9, 24]. In the present study, a significantly positive correlation was demonstrated between CLUE score and W/D ratio. This finding suggests that other than being non-invasive and easily accessible, assessment of EVLW with CLUE score is relatively as reliable as the evaluation of EVLW with W/D ratio. Thus, the additional evidence of CLUE’s potential is applied during donor procurement and EVLP keeping it in line with previous porcine CLUE and W/D ratio studies [9, 19, 24, 28]. Interestingly, in prior studies, CLUE has additionally been applied to rejected human lungs demonstrating a similar significant correlation between CLUE score and W/D ratio [9, 24]. This alludes further to the advantage of implementing the CLUE method in donor lung management.

EVLP has become a cornerstone method to sufficiently evaluate marginal donor lungs before transplantation/rejection [1–3, 22, 35]. Among others, lung parameters such as dynamic compliance and PVR are monitored during the EVLP in order to assess the real-time donor lung viability [22, 35]. Interestingly, some studies have cautiously indicated an association between these parameters and EVLW accumulation, while others have disputed this relationship with robust evidence of both assertions yet to be explored [36–38]. Nonetheless, the consensus among pulmonary specialists and EVLP experts is that dynamic compliance and PVR are fundamental variables to assess a lungs’ functionality and viability [22, 35]. In the present study, a significantly negative and a significantly positive correlation was demonstrated between CLUE score and dynamic compliance and PVR, respectively. These findings indicate that the trend of the CLUE score aligns effectively with the course of dynamic compliance and PVR development thus accentuating the essential ability of the CLUE method to be used in the setting of lung procurement and EVLP. No other study has assessed the relationship between these two parameters and CLUE score. However, one study demonstrated that the CLUE score has a sensitivity of 100% and specificity of 86% compared to dynamic compliance and PVR for LTx suitability evaluation and thereby endorsing the CLUE method to be highly accurate compared to conventional parameters for lung suitability assessment [9]. Considering this, routine application of CLUE during EVLP will encourage accurate real-time lung suitability decision and prompt early intervention to avoid further lung deterioration—e.g. immediate help deciding the EVLP approach (supine vs prone/double vs single) or clamping of the lobe bronchus with severe EVLW thus preventing contamination to viable lung lobes, which may be eligible for transplantation [24].

CLUE is a recent developed method with consequential limited but promising evidence [9, 24, 25]. Nevertheless, relying solely on CLUE as a single parameter to evaluate donor lung for LTx suitability can be misleading and may result in adverse outcomes after LTx [39, 40]. A more robust approach such as the ‘Comprehensive Lung Evaluation EVLP score’ may help assess donor lungs more sufficiently in a setting of EVLP and donor management with an established CLUE method as an incorporated paramount tool in decision-making of the best suitable donor lung for LTx [39, 40].

This study has several limitations. First, the lung tissue sample for W/D ratio of each corresponding CLUE point/image was not performed. Thus, the comparison of different CLUE grades and the correlation of CLUE point score with each CLUE point W/D ratio could not be analysed. The fact that harvested donor lungs are observed with marked heterogeneous distribution of EVLW within and between the lobes, this analysis would have given a more précis and ‘localized’ picture of CLUE grade and CLUE point score in relation to W/D ratio for each lobe. As a result, CLUE would have been strengthened if a relationship could be demonstrated with high and low EVLW content lobes calculated by CLUE score and subsequent W/D ratio as demonstrated in a previous CLUE study [24]. Second, a general correlation of CLUE score and P/F ratio was not analysed as it was deemed to demonstrate a biased picture of the lung due to the significant EVLW heterogeneity among the lobes. For the same reason, the correlation of CLUE score and PO_2_ was not considered accurate. Third, no blood samples from isolated pulmonary veins were taken, thus correlation of lobe score with the lobe P/F ratio or PO_2_ could not be tested. Furthermore, a small sample size with no power calculation in addition to the use of the porcine lung model limits the interpretation of the study results. A larger multicentre human donor lung study might be necessary to identify the CLUE score threshold defining the cut-off for LTx suitability—e.g. presented by a percentage increase in CLUE score value. Added to this point, the CLUE score alone cannot stand as a single parameter to assess LTx suitability. Lastly, lungs were not flushed with retrograde perfadex flush, which may have had an adverse effect on EVLW formation.

In conclusion, the CLUE technique is reproducible and feasible for monitoring EVLW in donor lungs after procurement and during EVLP using the porcine model. CLUE score correlated significantly with lung weight, W/D ratio, dynamic compliance and PVR suggesting the accuracy of measurement between these parameters and CLUE score. However, the transplant suitability of a donor lung may not depend solely on CLUE evaluation.

## Supplementary Material

ezae428_Supplementary_Data

## Data Availability

The raw data supporting the conclusions of this article will be made available by the authors upon reasonable request.

## ABBREVIATIONS

CLUE: direCt Lung Ultrasound Evaluation
CT: Computed tomography
EVLP: *Ex vivo* lung perfusion
EVLW: Extravascular lung water
LTx: Lung transplantation
P/F: Fraction of inspired oxygen
PiCCO: Pulse index continuous cardiac output
PO_2_: Partial pressure of oxygen
PVR: Pulmonary vascular resistance
T-LUS: Transthoracic lung ultrasound
W/D: wet-to-dry
