# Supplementary material for: Feasibility and accuracy of DireCt Lung Ultrasound Evaluation technique to monitor extravascular lung water in porcine lungs
Source: Eur J Cardiothorac Surg. 2024 Dec 10;67(1):ezae428. doi: 10.1093/ejcts/ezae428 (PMC11702978; doi:10.1093/ejcts/ezae428)

**Appendix**

**A: Inter-class coefficient for 2 raters of the CLUE images:**

icc(Inter_class_coeficcient_, model = "twoway",

+ type = "agreement", unit = "single")

Single Score Intraclass Correlation

Model: twoway

Type : agreement

Subjects = 36

Raters = 2

ICC(A,1) = 0.862

F-Test, H0: r0 = 0 ; H1: r0 > 0

F(35,2.52) = 35.1 , p = 0.013

95%-Confidence Interval for ICC Population Values:

0.113 < ICC < 0.96

**B: Lung Harvest, Preservation, and preparation for EVLP**

Pig anaesthesia: Animals were pre-medicated with zoletil (Zoletil^®^ Vet, Virbac, Denmark) (0.14 ml/ kg). General anaesthesia was achieved with 2,5% sodium pentobarbital (Pentothal, MTC Pharmaceuticals, Cambridge, Canada) 5mg/kg intravenous (i.v.). Anaesthesia was maintained with propofol (Propolipid^®^ 10 mg/ml, Fresenius Kabi AB, Uppsala, Sweden) 15 mg/kg/h i.v. and fentanyl (50 µg/ml, Hameln Pharma plus GMBH, Hameln, Saksa/Germany) 15 μg/kg/min i.v. Animals were intubated with a number 8 endotracheal tube (Portex, Sims, Markham, Canada) through the mouth.

Pig mechanical ventilation: Ventilation was performed with a volume-controlled ventilator (Servo Ventilator, Siemens-Elema Ab, Sweden). The fraction of inspired oxygen (FiO_2_) was 50%. Respiratory rate was adjusted to an end-tidal CO_2_ of 5-5.5 kPa. Tidal volume was set at 6-8 mL/kg and positive end expiratory pressure (PEEP) at 5 cm H_2_O. Baseline arterial blood gas measurements were obtained before sternotomy.

Harvest and Preservation: After sternotomy, thymus removal and pericardium opening, the superior and inferior vena cava were encircled with silk ties. A bolus of 15.000 IE Heparin (LEO Pharmaceutical, Copenhagen, Denmark) was injected i.v. A 21 French cannula was inserted into the main pulmonary artery (PA) and secured with a 4-0 prolene (Ethicon, Peterborough, Canada) purse-string suture. Before clamping the aorta, autologous whole blood was drained from the superior vena cava using 32 F venous cannula into a reservoir bag (TransMedic Inc.) for priming the EVLP. Upon arrythmia, aorta was clamped, the superior and inferior vena cavae were ligated and the heart was arrested by initiation of in situ antegraded flush with 2 litres of cold low-potassium dextran glucose preservation (LPDG) solution (Perfadex, XVIVO Perfusion AB, Gothenburg, Sweden). Left atrial appendage was transected to relieve the heart and the lungs. Ventilation was continued throughout the extraction of the heart-lung block. The trachea was clamped with the lungs inflated with a sustained airway pressure of 15 cm H_2_O. After removal of the heart-lung block, the lungs were weighted and stored in cold saline at 4-8°C for 2 hours. The collected autologous blood was added 10.000 U Heparin (Hepalean, Leo Pharma Inc. Ajax, Canada) and washed in a cell saver (Haemonetics model no. 2005) to collect washed red cells.

Preparation: The heart was excised from the heart-lung block. The remnant of the left atrium was trimmed to be wide open to prevent pulmonary vein outflow obstruction and to keep left atrium pressure near 0 mmHg. A cannula was sewn to the pulmonary artery (PA) with a 4-0 mono-filament suture. A sized-matched silicon tube was secured to trachea with silk ties. After the lungs were connected to EVLP system, a temperature probe was sutured inside the left atrium. All ex vivo lung perfusion was performed in antegrade fashion.

**C: EVLP procedure**

Priming: Apart from 2 litres of STEEN solution (XVIVO Perfusion AB), Heparin 15.000 IE (LEO Pharmaceutical, Copenhagen, Denmark), Meropenem 100mg, Methylprednisolone 1g and salvaged blood cells 500-800ml (to a haematocrit of > 10%) were used for priming. If needed, perfusate gasses were corrected on hourly basis as following: pH was maintained between 7.35 - 7.45 with 1 ml of isotonic trometamol (Addex-THAM 20ml, Fresenius Kabi AB, Uppsala, Sweden) for every unit below zero in base excess (12). Calcium was maintained > 3 mmol/L with Calcium Gluconate 10ml. Glucose was maintained > 5 mmol/L with Glucose Fresenius Kabi 100mg/ml.

All lung parameters were recorded from the EVLP- and respiratory monitors.

**D: Medians of each flow before and after EVLP**

| **40%** | | | |
| --- | --- | --- | --- |
|  | **Before (Median, IQR)** | **After (Median, IQR)** | **P-value** |
| Lung weight (gram) | 495 (475-530) | 961 (814-1041) | .002 |
| Wet-to-dry ratio | 0.673 (0.529-1.91) | 5.9 (5,74-6,4) | .004 |
| Dynamic compliance, ml/cm H_2_O | 36 (28.5-40) | 3 (2-4.75) | .002 |
| Pulmonary vascular resistance, dyn/sec/cm^2^ | 556 (522-700) | 1132 (955-1253) | .0008 |
| **80%** | | | |
| Lung weight (gram) | 456 (433-477) | 915 (767-995) | .002 |
| Wet-to-dry ratio | 0.914 (0.50-1,33) | 6.00 (5,77-6,12) | .002 |
| Dynamic compliance, ml/cm H_2_O | 32 (25.7-43.4) | 3 (2-4) | .008 |
| Pulmonary vascular resistance, dyn/sec/cm^2^ | 527.5 (412 - 576) | 693 (582 - 705) | .148 |
| **100%** | | | |
| Lung weight (gram) | 503 (464-534) | 979 (911-1005) | .002 |
| Wet-to-dry ratio | 1.16 (0.99-1.46) | 6.05 (5.9-6.07) | .002 |
| Dynamic compliance, ml/cm H_2_O | 46 (37.2-50) | 12 (8.2-14) | .004 |
| Pulmonary vascular resistance, dyn/sec/cm^2^ | 361 (323-439) | 1030 (717-1159) | .004 |
| *IQR*, Interquartile range | | | |

**E: Medians of each surface in each flow before and after EVLP**

| **40%** | | | |
| --- | --- | --- | --- |
|  | **Before (Median, IQR)** | **After (Median, IQR)** | **P-value** |
| CLUE score of anterior line | 1.5 (1.1-1.9) | 2.7 (2.2-2.7) | .01 |
| CLUE score of posterior line | 1.2 (1.0-1.8) | 3.7 (2.9-4.2) | .004 |
| CLUE score of lateral line | 1.6 (1.3-1.9) | 2.7 (24-2.9) | .04 |
| CLUE score of diaphragm line | 2.1 (1.6-3.2) | 3.3 (3-4.1) | .12 |
| **80%** | | | |
| CLUE score of anterior line | 1.2 (1.2-1.8) | 1.9 (1.8-2.3) | .1 |
| CLUE score of posterior line | 2.1 (1.8-2.6) | 3.6 (3.3-3.9) | .004 |
| CLUE score of lateral line | 1.1 (1.0-1.2) | 2.3 (2-2.7) | .004 |
| CLUE score of diaphragm line | 1.2 (1-1.8) | 4 (3.6-4.7) | .01 |
| **100%** | | | |
| CLUE score of anterior line | 1.3 (1.2-1.5) | 3.2 (2.8-3.4) | .008 |
| CLUE score of posterior line | 1.5 (1.2-1.9) | 3.6 (2.7-3.6) | .01 |
| CLUE score of lateral line | 1.1 (1-1.2) | 3.3 (2.7-3.5) | .004 |
| CLUE score of diaphragm line | 1.8 (1.6-2) | 3.4 (3-3.6) | .007 |
| *IQR*, Interquartile range | | | |

**F: Correlation of CLUE score with each variable in each EVLP flow group**

**40% EVLP flow**


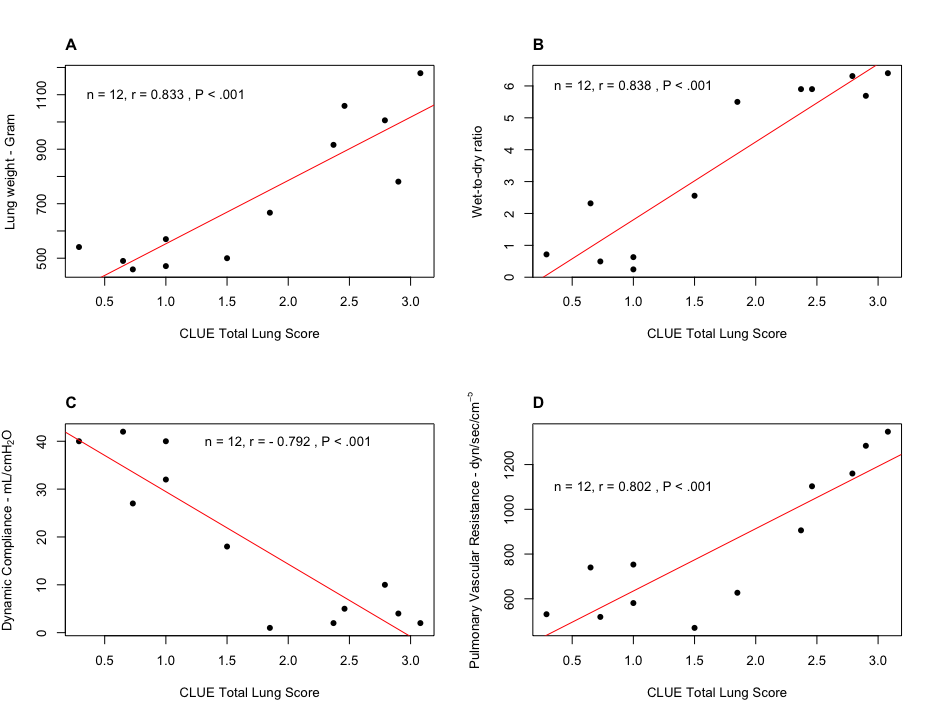


**80% EVLP flow**


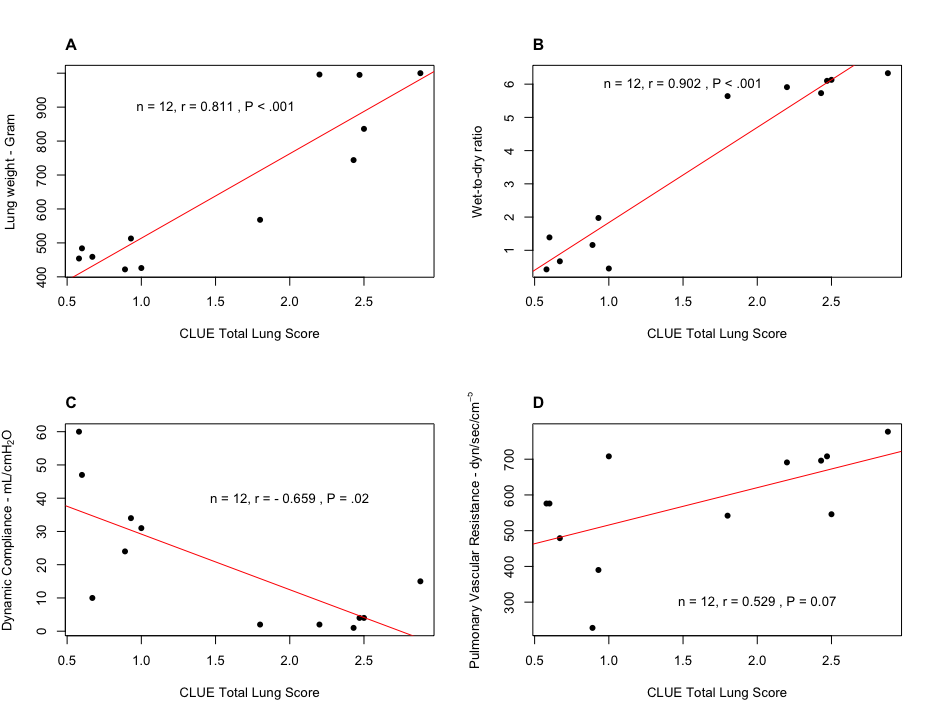


**100% EVLP flow**


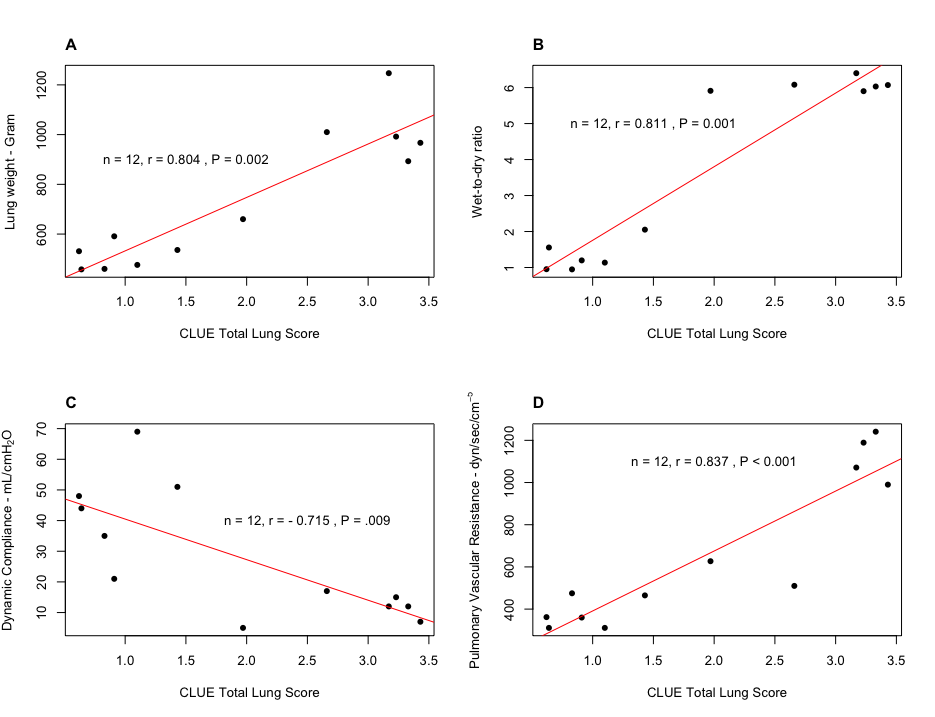

Supplement: ezae428_Supplementary_Data [file ezae428_supplementary_data.docx]
